# Aspernolide A Inhibits the Proliferation of Human Laryngeal Carcinoma Cells through the Mitochondrial Apoptotic and STAT3 Signaling Pathways

**DOI:** 10.3390/molecules24061074

**Published:** 2019-03-19

**Authors:** Chang Liu, Hong Liu, Yanzhang Wen, Huiqi Huang, Ji Hao, Yibing Lv, Rui Qin, Xinzhou Yang

**Affiliations:** 1 College of Life Sciences, South-Central University for Nationalities, Wuhan 430074, China; liuchang19941129@163.com (C.L.); 3032307@mail.scuec.edu.cn (H.L.); 2 School of Pharmaceutical Sciences, South-Central University for Nationalities, Wuhan 430074, China; 15172435677@163.com (Y.W.); Hhuiqi@hotmail.com (H.H.); 13618615220@163.com (J.H.); m13525371866@163.com (Y.L.)

**Keywords:** aspernolide A, *camptotheca acuminata decne*, *cladosporium cladosporioides*, anticancer, laryngeal cancer, apoptosis, mitochondrial pathway, STAT3

## Abstract

Aspernolide A, a butyrolactone secondary metabolite, was purified from the endophytic fungus *Cladosporium cladosporioides* derived from roots of *Camptotheca acuminata* Decne. In this study, the antitumor activity and mechanisms of aspernolide A on human laryngeal cancer Hep-2 and TU212 cells were studied by MTT (3-(4,5-Dimethylthiazol-2-yl)-2,5-diphenyltetrazolium bromide) assay, morphological observation and Western blotting. The results showed that aspernolide A significantly inhibited the proliferation of Hep-2 and TU212 cells in dose- and time-dependent manners. Morphological changes of apoptotic cells could be observed under an inverted microscope, such as irregular margins, decreased adherence ability and chromatin condensation. The expressions of Bax, Caspase-9, Caspase-3 and PARP (poly ADP-ribose polymerase) increased with the increase of dosage while Bcl-2 decreased, suggesting that the apoptotic mechanism might be related to the mitochondrial apoptotic pathway. Moreover, the expression of the phosphorylation of STAT3 decreased with the increase of dosage, suggesting that the apoptotic mechanism might be related to the STAT3 signaling pathway. All these conclusions indicated that aspernolide A has the potential anti-laryngocarcinoma effects.

## 1. Introduction

Laryngeal cancer is one of the most common malignant tumors of the head and neck [1]. The incidence of laryngeal squamous cell carcinoma ranks third among the tumors of the head and neck [2]. The European Cancer Observatory data for the European Union indicate that laryngeal squamous cell carcinoma (LSCC) is the 13th most common neoplasm in men [3]. The treatment of laryngeal cancer has undergone tremendous changes in the past few years. At present, therapy is determined by age, performance status, stage of disease and the tumour location [4]. Total laryngotomy is considered to be the most effective method for the treatment of laryngeal cancer, but costs a large amount, which brings some adverse sequelae to patients, such as hypothyroidism [5]. Although early-stage laryngeal cancer is often curable with surgery or radiotherapy, for the majority of patients with the advanced disease, the outcome has not improved dramatically in the last two decades despite therapeutic advances [6]. In order to further to improve survival rates and treatments, it is important to better understand the mechanisms of carcinogenesis. Despite the fact that considerable efforts have been made in recent years, the molecular mechanism involved in the initiation and progression of laryngeal carcinoma remains largely unknown. Consequently, radiotherapy and chemotherapy have proven to be the most frequently used methods for the great majority of cancerous persons. Hence, finding the appropriate anti-carcinogen has turned into a top priority.

Natural products are important sources of new compounds of various structures with different biological activities, and some reports indicate that natural products and their derivatives have irreplaceable effects on the field of drug discovery [7]. Natural products are the main source of high-efficiency and low-toxicity anticancer agents. For instance, camptothecin is the secondary metabolic product of *Camptotheca acuminata* Decne, and it is widely used in the treatment of liver cancer, gastric cancer, bladder cancer and leukemia [8]. Taxol is considered to be one of the most effective anticancer drugs in the world [9]. A taxol-producing endophytic fungus *Taxomyces andreanae* was isolated from *Taxus brevifolia*, and its taxol content was 24–50 ng/L, which showed a new way to produce taxol [10]. The uses of natural products of cancer chemotherapy have provided more possibilities for the treatment of cancer and have a high value of development and utilization. Therefore, it is feasible to find new compounds from natural resources to fight human laryngeal carcinoma, which can meet the growth needs of the development of chemotherapy.

In the present study, aspernolide A, a butyrolactone produced from an endophytic fungus *Cladosporium cladosporioides* derived from the roots of *Camptotheca acuminata* Decne was isolated and purified by means of repeated column chromatography. Research by others showed that aspernolide A was tested against soybean lipoxygenase, and five cell lines displayed weak cytotoxicity against H460, ACHN, Calu, Panc1 and HCT116 cell lines (IC_50_ > 88, >103, >147, >130, >121 µM, respectively) [11,12]. However, the cytotoxicity of aspernolide A against laryngeal cancer cell lines has not been elucidated. In the current research, we evaluated the cytotoxic activity of aspernolide A and preliminarily elucidated the antitumor mechanism of aspernolide A on Hep-2 and TU212 cell lines.

## 2. Results

### 2.1. Effects of Aspernolide A on Cell Viability in Hep-2 and TU212 Cells

**Figure 1:**
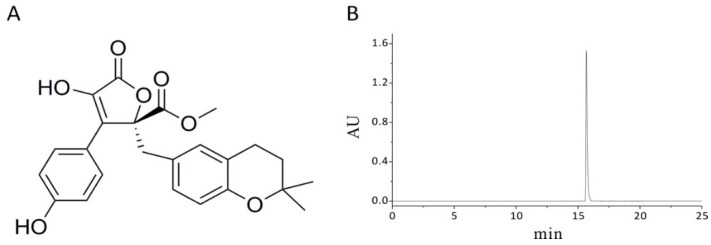
The chemical structure and purity of aspernolide A. (**A**) Chemical structure of aspernolide A; (**B**) HPLC chromatogram of aspernolide A.

The endophytic fungus *Cladosporium cladosporioides* was isolated and determined from the roots of *Camptotheca acuminate*. Afterwards, aspernolide A was purified by repeated column chromatography from the fermented rice medium of *C. cladosporioides*. The chemical structure and HPLC chromatogram of aspernolide A are shown in Figure 1.

**Figure 2:**
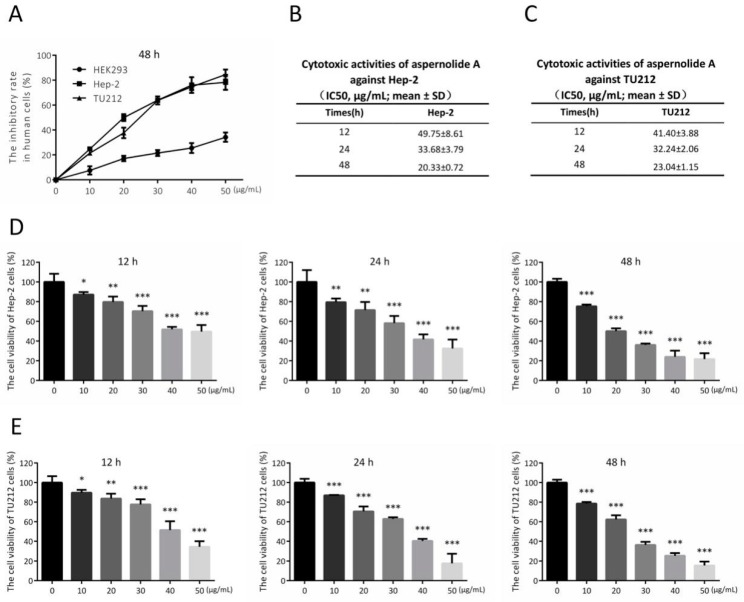
Aspernolide A provoked the apoptosis of human laryngeal carcinoma cells. (**A**) Cell viability and cytotoxicity was determined using the MTT assay. (**B**,**C**) IC_50_ values of Hep-2 and TU212 cells in 12, 24 and 48 h were shown. (**D**,**E**) Hep-2 and TU212 cells were treated with indicated concentrations of aspernolide A. After 12, 24 and 48 h, cell apoptosis was examined by MTT. (* *p* < 0.05, ** *p* < 0.01 or *** *p* < 0.001).

We investigated the anti-proliferation of aspernolide A in Hep-2 and TU212 cells using MTT assay. HEK293 cells, a human non-cancerous kidney cell line, was also incubated under the same conditions as human laryngeal carcinoma cells to evaluate the toxic impacts. It was observed that aspernolide A has a better inhibitory effect on Hep-2 cells in 24 h (IC_50_ = 33.68 µg/mL) and 48 h (IC_50_ = 20.33 µg/mL) than 12 h. Aspernolide A also showed a better inhibitory effect on TU212 cells in 24 h (IC_50_ = 32.24 µg/mL) and 48 h (IC_50_ = 23.04 µg/mL) than 12 h. In these results (Figure 2), it can be seen that, with the increasing doses of aspernolide A, the cell viability of Hep-2 and TU212 cells gradually decreased. In addition, no distinct antiproliferative activity against HEK293 cells was observed in 48 h (IC_50_ > 50 µg/mL). Moreover, the dose- and time-dependent chart showed that aspernolide A inhibited the growth of the human laryngeal carcinoma cell lines in dose- and time-dependent manners.

### 2.2. Effects of Aspernolide A on Cell Migration and Colony Formation in Hep-2 and TU212 Cells

**Figure 3:**
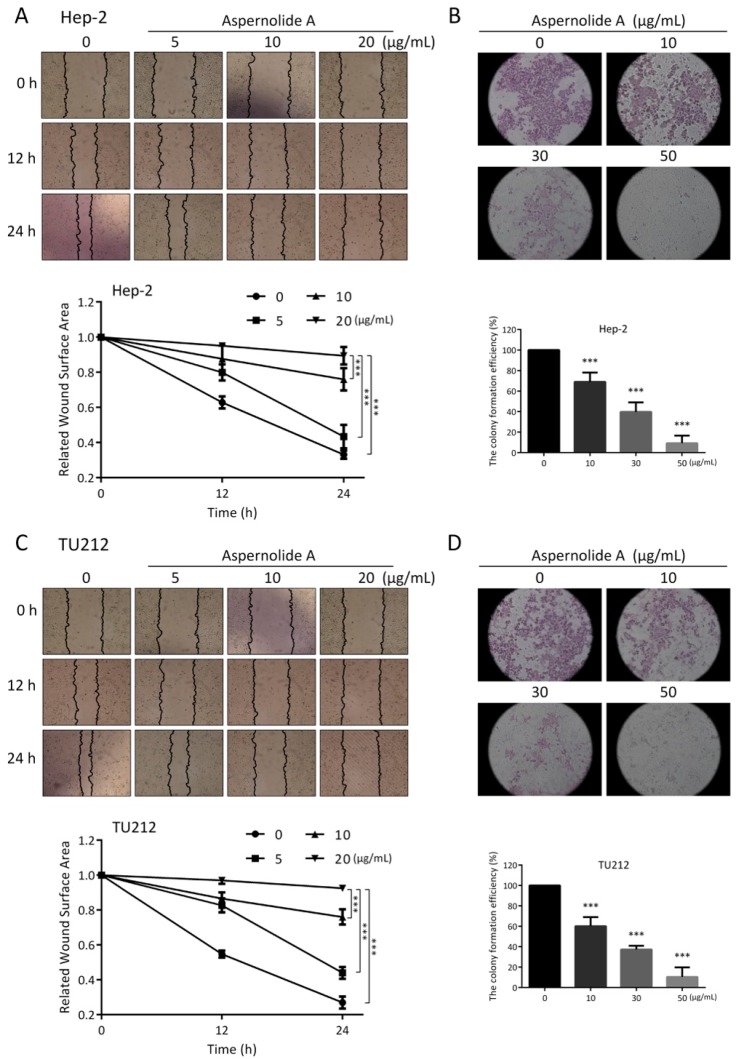
Cell migration and colony-forming in Hep-2 and TU212 cells with aspernolide A. (**A**,**C**) Representative images of scratch assay and its counting results. (**B**,**D**) Representative images of colony-forming assay and their counting results. (* *p* < 0.05, ** *p* < 0.01 or *** *p* < 0.001).

Collective cell migration is a sign of cancer invasion. Quantitative wound healing is an effective way to evaluate the migration ability of cancer cells. In our study, the effect of aspernolide A on the migration of laryngeal cancer Hep-2 and TU212 cells was determined by wound healing test. The scratch assay (Figure 3A,C) showed that aspernolide A (5, 10, 20 µg/mL) significantly reduced cell migration after 12 h and 24 h in Hep-2 and TU212 cells. The relative wound surface areas of 10 and 20 µg/mL groups were more than 0.8 at 12 h and more than 0.6 at 24 h, while the inhibition of Hep-2 and TU212 cell proliferation at those concentrations was inconspicuous.

Additionally, crystal violet is an alkaline dye that can combine with nucleic acid in the nucleus to dye the nucleus blue–purple. The colony formation (Figure 3B,D) of Hep-2 and TU212 cells was significantly suppressed by aspernolide A in a concentration-dependent manner compared to the control ones. The colony formation efficiency of 30 and 50 µg/mL groups was less than 50% in Hep-2 cells and less than 40% in TU212 cells. These results suggested that aspernolide A restrained Hep-2 and TU212 cell proliferation, particularly at a high concentration.

### 2.3. Effects of Aspernolide A on Morphological Changes and Apoptosis in Hep-2 and TU212 Cells

**Figure 4:**
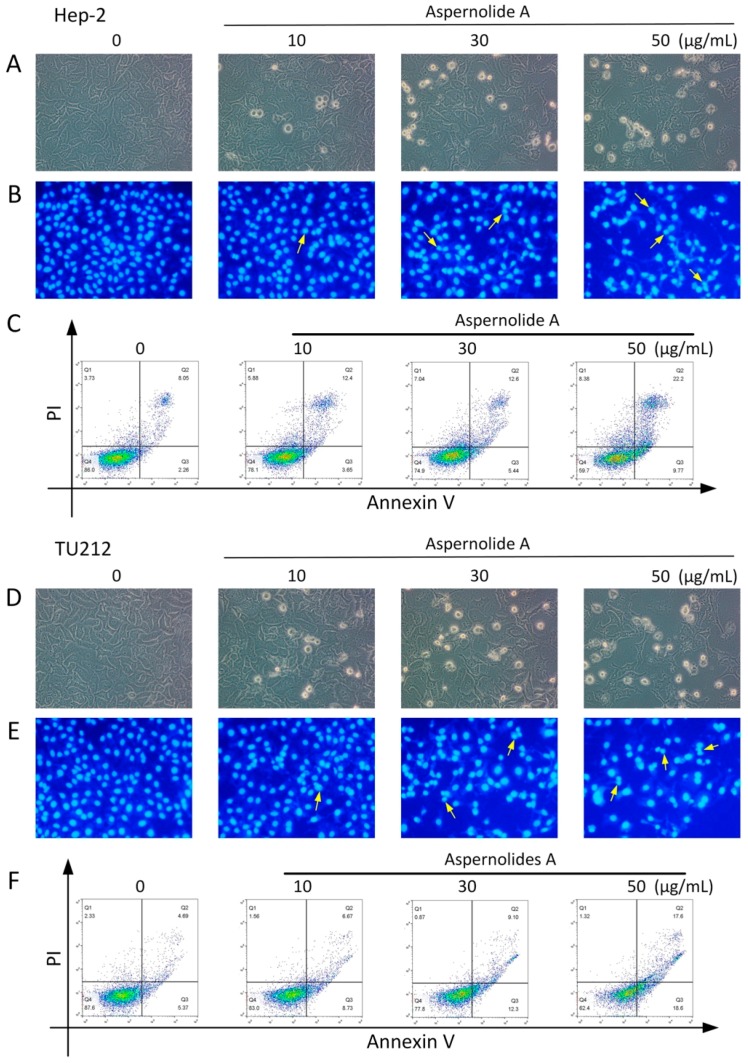
Observation of morphological changes and apoptosis in Hep-2 and TU212 cells with aspernolide A. (**A**,**D**) The morphology of Hep-2 and TU212 cells were photographed under an inverted-phase contrast microscope (80×). (**B**,**E**) The morphology of Hep-2 and TU212 cells were photographed under a fluorescence microscope (80×) with Hoechst 33258. (**C**,**F**) Hep-2 and TU212 cells were treated with aspernolide A for 12 h, then stained with Annexin V/PI staining and then evaluated by flow cytometry.

The morphological changes of Hep-2 and TU212 cells treated with different concentrations of aspernolide A were observed under an inverted-phase contrast microscope. It was observed that, with the increase of dosage, adherent cells gradually decreased, floating cells gradually increased, and cells almost completely fell off at a high dosage (Figure 4A,D). After staining (Figure 4B,E), compared with the drug group, the cells in the blank group were intact and uniformly colored. Apoptotic phenomena were observed in the drug group with chromatin condensation, partitioning into blocks, and appearing as bright apoptotic bodies, especially in the high-dose group.

In order to examine whether aspernolide A could induce apoptosis after 12 h in Hep-2 and TU212 cells, cells were stained with Annexin V-fluorescein isothiocyanate (FITC) and analyzed by flow cytometry. The experimental data were taken with Annexin V as the horizontal axis and propidium iodide (PI) as the vertical axis. The left upper quadrant shows mechanically damaged cells, the right upper quadrant shows late apoptotic or necrotic cells, the left lower quadrant shows negative normal cells, and the right lower quadrant shows early apoptotic cells. The percentage of apoptotic Hep-2 cells was significantly higher after incubation with different concentrations (10, 30, 50 µg/mL) of aspernolide A (Q2 + Q3: 16.05%; 18.04%; 31.97%) than that of the control group (Q2 + Q3: 10.31%) (Figure 4C). The percentage of apoptotic TU212 cells was also significantly higher after incubation with different concentrations (10, 30, 50 µg/mL) of aspernolide A (Q2 + Q3: 11.19%; 21.40%; 36.20%) than that of the control group (Q2 + Q3: 10.06%) (Figure 4F).

### 2.4. Effects of Aspernolide A on the Mitochondrial Apoptotic Pathway in Hep-2 and TU212 Cells

**Figure 5:**
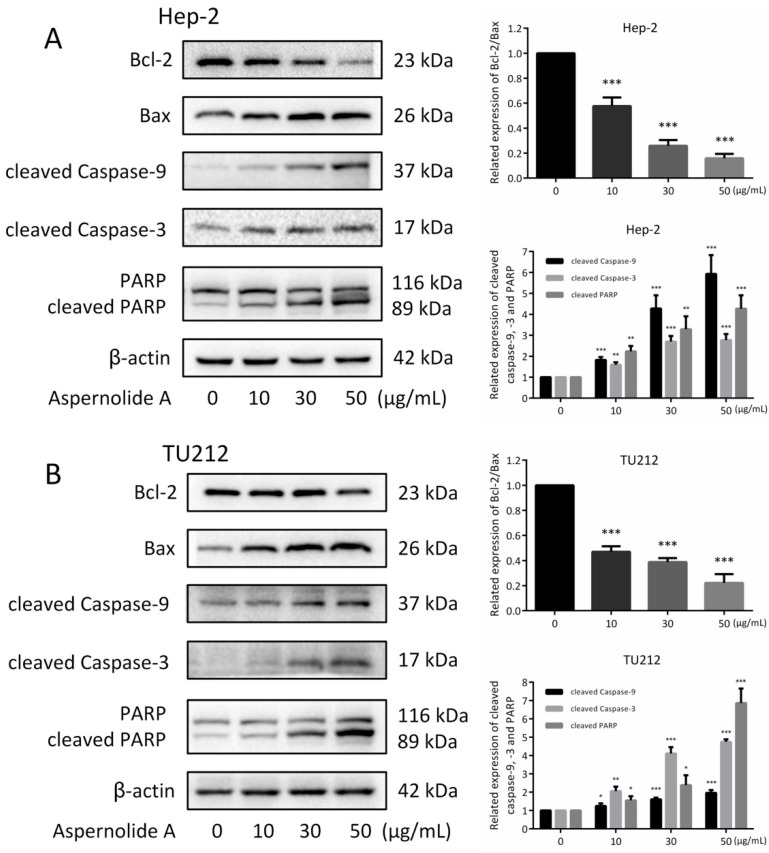
Effects of aspernolide A on the apoptosis regulatory proteins of laryngeal cancer cells. (**A**,**B**) The related expressions of Bcl-2/Bax, cleaved caspase-9, cleaved caspase-3 and cleaved PARP in Hep-2 and TU212 cells were detected by Western blotting. β-actin was used as a control. (* *p* < 0.05, ** *p* < 0.01 or *** *p* < 0.001).

In the process of apoptosis, cytochrome C in mitochondria is released into the cytoplasm, which can down-regulate the expression of downstream protein Bcl-2 and up-regulate the expression of Bax. Similarly, caspases also play an important role in the apoptotic signaling pathway network. The pathway of apoptosis depends on the activation of caspase, which is the final execution of apoptosis [13]. In order to determine the mechanisms of apoptosis induced by aspernolide A in Hep-2 and TU212 cells, we investigated the expression of Bcl-2, Bax, cleaved caspase-9, cleaved caspase-3 and cleaved PARP using Western blotting analysis. Western blotting was used to verify whether aspernolide A activated the mitochondrial apoptosis-related proteins. Treatment with different concentrations (10, 30, 50 µg/mL) of aspernolide A in Hep-2 and TU212 cells increased the expressions of cleaved caspase-3, caspase-9 and PARP (Figure 5). In addition, aspernolide A increased the expression of Bax and decreased the expression of Bcl-2 in Hep-2 and TU212 cells.

### 2.5. Effect of Aspernolides A on STAT3 Signaling Pathway in Hep-2 and TU212 Cells

**Figure 6:**
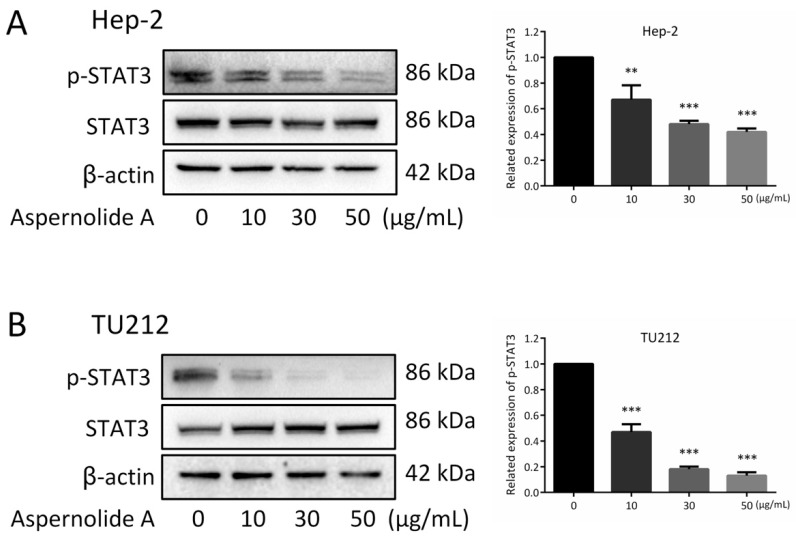
Aspernolide A repressed STAT3 activity in Hep-2 and TU212 cells. (**A**,**B**) The related expressions of p-STAT3 and STAT3 were detected in Hep-2 and TU212 cells by Western blotting. β-actin was used as a control. (* *p* < 0.05, ** *p* < 0.01 or *** *p* < 0.001).

STAT3 (Signal transduction and activator of transcription 3) is the aggregation point of many single gene signaling pathways, is activated at high frequency in a wide diversity of cancers, and is a promising molecular target for cancer treatment [14]. Hence, the expression of STAT3 was investigated. As shown in Figure 6, aspernolide A dose-dependently repressed the basal phosphorylation of STAT3 (p-STAT3) but did not inhibit the protein levels of total STAT3 in suppressing Hep-2 and TU212 cell proliferation.

**Figure 7:**
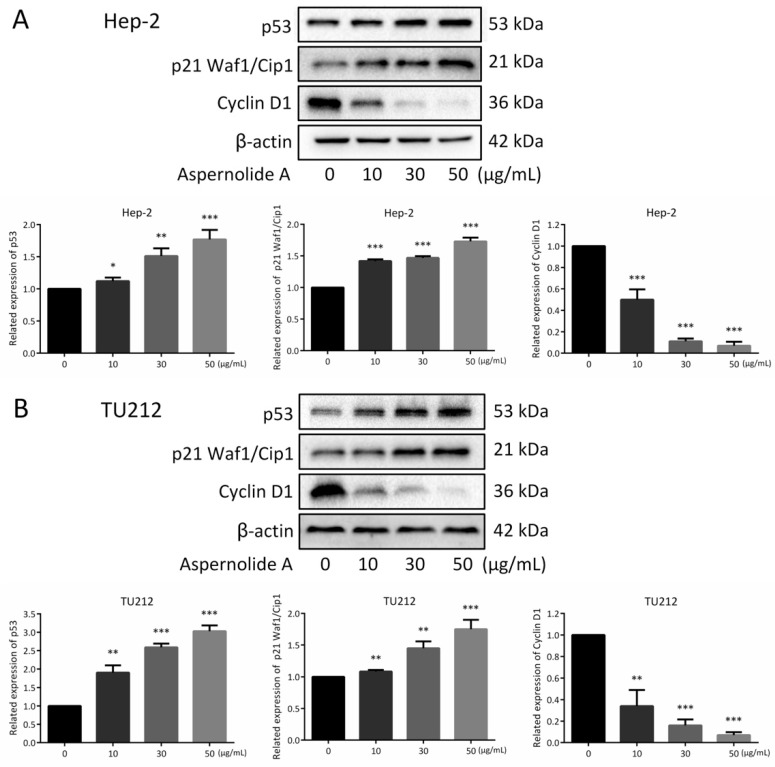
Effects of aspernolide A on the cell cycle related proteins of laryngeal cancer cells. (**A**,**B**) The related expression of p53, p21 Waf1/Cip1 and Cyclin D1 were detected in Hep-2 and TU212 cells by Western blotting. β-actin was used as a control. (* *p* < 0.05, ** *p* < 0.01 or *** *p* < 0.001).

Moreover, blocking STAT3 in cancer cells can up-regulate the expression of p53, leading to the p53-mediated apoptosis of cancer cells [14], and initiate the transcription of many downstream genes, including p21 Waf1/Cip1. Then, p21 Waf1/Cip1 can bind with cyclin-CDK (cyclin-dependent kinases) complexes to inhibit the activity of cells and arrest the cell cycle. In the case of serious DNA damage, p53 will induce the expression of apoptotic factors and cause programmed cell death [15,16]. Additionally (Figure 7), aspernolide A increased the expressions of p53 and p21 Waf1/Cip1, and then decreased the expression of Cyclin D1.

## 3. Discussion

Among the most frequent malignant tumors in the world, laryngeal cancer has not shown an increase in 5-year survival rates over the past 30 years [17]. The increasing use of chemo- and radiotherapy and conservative surgery to preserve organs and their functions has probably led to a better quality of life in patients with laryngeal cancer, but has definitely failed to improve survival, which remains the primary aim [17]. Therefore, understanding the formation and characteristics of cancer cells is not only helpful to understanding the regulation of cell proliferation, differentiation and apoptosis and its molecular mechanism, but also to solve the problems of human health.

In recent years, more and more attention has been focused on natural products because of their low toxicity in cancer treatment [18]. In all kinds of natural resources, plants have been extensively explored for their phytochemistry and pharmacology; however, only a small proportion of known microbial secondary metabolites have been studied for their therapeutic potential [19]. Therefore, the research of drugs based on microbial natural products is becoming more valuable, especially in terms of their anti-cancer efficacy.

Previous studies have shown that aspernolide A weakly inhibited the activities of several kinds of human carcinoma cell lines [12], whereas its possible antitumor activities of laryngeal cancer cell lines have not been as well-studied. In this paper, we showed that aspernolide A inhibited the growth of human laryngeal cancer Hep-2 and TU212 cells in time- and concentration-dependent manners (Figure 2). In the scratch assay (Figure 3A,C), compared with the control group, the wound surface area of the aspernolide A-treated group had a worse recovery ability in time- and concentration-dependent manners. Simultaneously, colony-forming experiments (Figure 3B,D) also proved that aspernolide A inhibited the growth of laryngeal cancer cells Hep-2 and TU212 in a concentration-dependent manner. As evidenced by the above results, aspernolide A exhibited significant antitumor activity.

There are two different forms of cell death: cell necrosis and cell apoptosis [20]. Necrosis is a process of rapid cell death caused by some external factors, such as high fever, physical and chemical damage, and it is a passive death [21]. Apoptosis is a process of programmed cell death, which is caused by a suicide program controlled by genes [22]. The present study displayed that the cell morphology changed obviously (Figure 4A,B,D,E): the cell edge was irregular, the adherence ability decreased, the chromatin condensed, the fluorescence became dense, and bright blue spots (apoptotic bodies) could be seen. Annexin V-FITC/PI double staining (Figure 4C,F) also confirmed that aspernolide A could induce apoptosis.

Chemotherapeutic drugs induce apoptosis by acting on different target proteins in different signaling pathways, such as the mitochondrial apoptotic pathway and JAK-STAT (Janus kinase-signal transducers and activators of transcription) signaling pathway [23]. The regulatory factors related to tumorigenesis mainly include extracellular signal molecules, signal receptors, intracellular signal transduction proteins and transcription factors, cell cycle regulatory proteins, DNA damage repair regulatory proteins and apoptotic proteins [24]. These regulatory factors are involved in various signaling pathways of cell life activities, and the imbalance of them will lead to the blockade of cell life activities, or the unrestricted rapid proliferation (canceration) or cell death [25]. In our study, we explored the possible molecular mechanisms of aspernolide A against laryngeal cancer. Western blotting was used to detect the expressions of five proteins (caspase-9, caspase-3, PARP, Bcl-2 and Bax) in Hep-2 and TU212 cells treated with different concentrations of aspernolide A. The results showed that the expressions of caspase-9, caspase-3 and PARP increased with the dose increasing, while the ratio of Bcl-2/Bax decreased (Figure 5). Caspases are widely expressed in various cells and can activate other proteases to initiate a protease cascade [25]. In this family, caspases are identified and categorized into initiators (caspase-2, -8, -9, -10), effectors (caspase-3, -6, -7), and inflammatory caspases (caspase-1, -4, -5) [26]. Caspase-9 is the apoptotic initiator in the upstream of cascade reaction. It can activate downstream apoptotic executing factors caspase-3 and PARP, and then induce apoptosis [27]. Bcl family proteins can regulate apoptosis by altering the permeability of mitochondrial extracorporeal membrane and affecting the release of mitochondrial membrane gap proteins [28]. Bcl-2 and Bax proteins are the two most important proteins in this family [29]. Therefore, we speculate that a similar mechanism may be involved in the growth inhibition of laryngeal cancer induced by aspernolide A.

**Figure 8:**
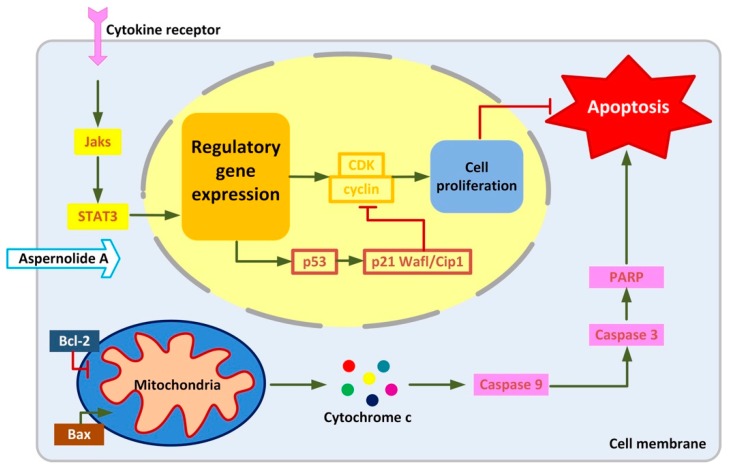
The mechanism of apernolide A exerting its efficacy in laryngeal cancer cells.

It can be seen from the results that blocking STAT3 in cancer cells can up-regulate the expression of p53, and the up-regulation of p53 can affect the expression of cyclin, leading to cell cycle arrest. The present research displayed that aspernolide A dose-dependently reduced the protein levels of p-STAT3 and Cyclin D1, and dose-dependently increased p53 and p21 Waf1/Cip1 (Figure 6 and Figure 7). STAT has dual functions of signal transduction and transcriptional activation, and the abnormal persistent activation of STAT3 signal has been found in human cancer cells [30]. In addition, some studies found that STAT3 and p-STAT3 were overexpressed in laryngeal cancer tissues, and both were localized in the cytoplasm and nucleus, and related to clinical stage and lymph node metastasis [31]. The results confirmed our hypothesis that treatment of aspernolide A inhibited the activity of STAT3 in laryngeal cancer, suggesting that the apoptosis of laryngeal cancer cells induced by aspernolide A may be caused by the inhibition of STAT3. Cyclin D1 is an important factor in promoting the transition from G0/G1- to S-phase, and p53 and p21 waf1/cip1 are the key factors in cell cycle progression. On the contrary, p53 and p21 waf1/cip1 are negative regulators of this transition [32,33]. Our results also proved that the antitumor activity of aspernolide A was related to p53, p21 Waf1/Cip1 and cyclin D1 proteins. The mechanism of apernolide A exerting its efficacy in laryngeal cancer cells can be seen in Figure 8.

## 4. Materials and Methods

### 4.1. Chemicals and Reagents

High performance liquid chromatography (HPLC)-grade solvents were used for chromatography and all the other chemicals were of analytical-reagent grade. HPLC-grade acetonitrile (MeCN) and methanol were purchased from Merck Chemical Company (Darmstadt, Germany). Sephadex LH-20 gel was obtained from GE Health Care (Uppsala, Sweden). The 2-NBDG assay kits were purchased from Cayman Chemical (Ann Arbor, MI, USA). Minimum Essential Medium-α (MEM-α) and fetal bovine serum (FBS, and antibiotics (100 U/mL penicillin and 100 μg/mL streptomycin)) were obtained from Hyclone (Logan, UT, USA).

### 4.2. General Experimental Procedures

Semi-preparative HPLC was carried out on a Waters 2535 HPLC fitted with a 2998 Photodiode Array Detector and a 2707 Autosampler (Waters, Milford, MA, USA). Separations were performed on Thermo C18 columns (5 μm, 10 × 150 mm) (Waters, Milford, MA, USA) and Phenomenex (5 μm, 20 × 150 mm). Direct injection ESIMS (Engmeering Services Information Management System) and LC-PDA-ESIMS (Ultra performance liquid chromatography-electrospray ionization mass spectrometry) analyses were recorded on a Waters ACQUITY SQD MS system (Waters, USA) connected to a Waters 1525 HPLC with a 2998 Photodiode Array Detector (Waters, USA). The NMR spectra were recorded on an AVANCE III 600 MHz spectrometer (Bruker BioSpin, Ettlingen, Germany). Optical rotations were recorded on a Rudolph Research Analytical Autopol IV Automatic Polarimeter (AutoFill, Hackettstown, NJ, USA). UV and IR spectra were recorded on a UH5300 spectrophotometer (Hitachi, Japan) and a Nicolet Magna FT-IR 750 spectrometer (Nicolet, Madison, USA), respectively.

### 4.3. Source and Isolation of Fungus

The roots of *Camptotheca acuminata* Decne were collected in June 2017 in the campus of South-Central University for Nationalities (SCUN), Wuhan, China. The microorganisms from roots of *C. acuminata* were isolated by four media. Culture media include *Czapek—Dox* medium, *Sabouraud* medium, *Cochrane 1* medium and PDA culture medium. Forty milligrams of penicillin and 40 mg streptomycin were added to 1 L medium. Based on the TLC analysis of secondary metabolites of endophytic fungi, an endophytic fungus was purified and a large amount of fermentation was carried out to obtain full secondary metabolites. The strain was identified as *Cladosporium cladosporioides*. The voucher specimen (No. L6-h1126J-2 51-2) is deposited in School of Pharmaceutical Sciences, SCUN, Wuhan, China.

### 4.4. Extraction and Isolation

The fermented rice medium of *C. cladosporioides* was extracted seven times with 80% EtOH. After the removal of solvent under reduced pressure, the crude extract (185.5 g) was suspended in 2 L water and then extracted with ethyl acetate four times to give an EtOAc extract (50.5 g). The EtOAc extract (50 g) was subjected to a middle pressure column chromatography (MPCC) with silica gel using a gradient solvent system of CH_2_Cl_2_-MeOH (200:1 → 100:1 → 50:1 → 30:1 → 20:1 → 1 0:1 → 5:1 → 1:1 → 0:1, *v*/*v*, containing 0.1% formic acid) to give six major fractions Fr 1 (2.5 g), Fr 2 (3.8 g), Fr 3 (6.3 g), Fr 4 (5.7 g), Fr 5 (7.5 g) and Fr 6 (4.4 g). Fr 5 (6.1 g) were subjected to a MPCC with silica gel (CH_2_Cl_2_-MeOH, 20:1-10:1-5:1-1:1-0:1, containing 0.1% formic acid) to afford four fractions Fr 5.1 (1.2 g), Fr 5.2 (2.1 g), Fr 5.3 (1.3 g), Fr 5.4 (0.4 g). Fr 5.3 (1.1 g) were purified by sephadex LH-20 CC (CH_2_Cl_2_/MeOH, 1:2, containing 0.1% formic acid) and then semipreparative HPLC (H_2_O /MeCN, 90:10 → 20:80, 25 min, containing 0.1% formic acid in both two phases) to yield aspernolide A (168 mg), it has been elucidated as aspernolide A by comparing its ^1^H- and ^13^C-NMR data (Figures S1 and S2 in Supplementary Material) with the reported literature [12].

### 4.5. Cell Culture

Human laryngeal cancer cell lines Hep-2 and TU212 and human embryonic kidney cell line HEK293 were obtained from American Type Culture Collection (ATCC). Hep-2 and TU212 cells were grown in DMEM medium (Sigma-Aldrich, St. Louis, MO, USA), supplemented with 10% fetal bovine serum, 1% penicillin and streptomycin (Hyclone, Logan, UT, USA). Cells were cultured in an incubator containing a humidified atmosphere with 5% CO_2_ at 37 °C.

### 4.6. MTT Assay

The MTT method was used to test the cytotoxicity of aspernolide A on laryngeal cancer of Hep-2 and TU212 cells. The cells were seeded in 96-well plates until the density was 1 × 10^5^ cells in each well and exposed to different concentrations of aspernolide A (0, 10, 20, 30, 40, 50 µg/mL). Following 12, 24 and 48 h of drug treatment, 100 µL MTT (5 mg/mL) was added to each well. After incubating for 30 min, the liquid in the wells was discarded and added 150 µL DMSO to each well. The OD values were measured at 562 nm with a spectrophotometer (Thermo Fisher Scientific Oy, Vantaa, Finland). Percentage of cell viability ratio = [1 − (OD_treatment group_ − OD_blank group_)/(OD_control group_ − OD_blank group_)] × 100% [34]. The inhibition rates and IC_50_ values were calculated by GraphPad Prism 6.0 and plotted.

### 4.7. Morphological Changes of Apoptosis

The cells were seeded in 6-well plates until the density was 2.5 × 10^6^ cells in each well and exposed to different concentrations of aspernolide A (0, 10, 30, 50 µg/mL). Following 24 h of drug treatment, under an inverted phase contrast microscope (Soptop ICX41, Ningbo, China), the morphology of the cells was photographed by the camera at 80× magnification. Shortly afterwards, 1.0 mL of stationary liquid (the ratio of methanol to acetic acid is three to one) was added to each well. After holding the solution in place for 15 min, the cells were stained with Hoechst 33258 for 15 min under dark conditions. Under a fluorescence microscope (excitation wavelength 350 nm and emission wavelength 460 nm, Soptop ICX41, Ningbo, China), the morphology of the cells was photographed by the camera at 80× magnification.

### 4.8. Scratch Assay

The cells were seeded in 6-well plates until the density was 2.5 × 10^6^ cells in each well and exposed to different concentrations of aspernolide A (0, 5, 10, 20 µg/mL). The plate surface was wounded with a sterile pipette tip (10 µL) to make the gap between the cells and washed with PBS to remove the detached cells. Following 0 h, 12 h and 24 h of drug treatment, under an inverted phase contrast microscope (Soptop ICX41, Ningbo, China), the scratch wounds of the cells were photographed by the camera at 16× magnification.

### 4.9. Colony Forming Test

The cells were seeded in 6 cm cell culture dishes until the density was 5.2 × 10^6^ cells in each dish and exposed to different concentrations of aspernolide A (0, 10, 30, 50 µg/mL). Following 14 days of drug treatment (change the solution twice a week), 1 mL 0.1% crystal violet was added to each plate. After staining for 10 min, the cells were washed twice with ultrapure water. Under a microscope (Soptop ICX41, China), the number of colonies containing 50 cells was calculated. And the plate colony formation efficiency was calculated using the formula: plate colony formation efficiency = (number of colony/number of cells inoculated) × 100% [35].

### 4.10. Annexin V-FITC Double Staining

The cells were seeded in 6-well plates until the density was 2.5 × 10^6^ cells in each well and exposed to different concentrations of aspernolide A (0, 10, 30, 50 µg/mL). Following 12 h of drug treatment, the cells were washed twice with PBS and centrifuged for 5 min. Then, 100 μL 1 × binding buffer was added to re-suspended cells and incubate with 5 μL of annexin V-fluorescein isothiocyanate (FITC) and 5 μL of propidium iodide (PI) at room temperature for 10 min in the dark. Before measurement, another 400 μL 1 × binding buffer was added. A flow cytometer (Guava easyCyte, Darmstadt, Germany) was used to explore the cell apoptosis.

### 4.11. Western Blotting

The cells were seeded in 10 cm cell culture dishes until the density was 13.7 × 10^6^ cells in each dish and exposed to different concentrations of aspernolide A (0, 10, 30, 50 µg/mL). Following 24 h of drug treatment, the cells were mechanically dissociated into single-cell suspension and the protein was extracted. After the denaturation of the protein, the protein concentrations were measured using a BCA (bicinchoninic acid) kit (Beyotime, Shanghai, China). SDS-PAGE gel electrophoresis was performed at 120 V voltage until bromophenol blue was terminated at the lower edge of the electrophoresis tank. After transferring for 1 h 20 min at a current of 300 mA, the membranes were incubated in 5% skim milk for 2 h at room temperature, and then the PVDF (polyvinylidene fluoride) membranes were washed in TBST (Tris Buffered saline Tween). Primary antibodies were applied overnight and secondary antibodies were applied for 2 h at room temperature. Under the gel imager (Bio-Rad, CA, USA), the protein on the PVDF membranes appeared.

### 4.12. Statistical Analysis

The data were expressed as mean ± SD for at least three separate experiments. GraphPad Prism 6.0 software was used for all statistical analyses. Statistical differences were analyzed by one-way analysis of variance (ANOVA) and statistical significance was determined as * *p* < 0.05, ** *p* < 0.01 or *** *p* < 0.001.

## 5. Conclusions

Cancer is one of the most fatal diseases in the world [36]. Drug therapy can regulate the rate of cell apoptosis and proliferation, thus killing cancer cells and maintaining the stability of the number of cells in tissues and organs and the natural regeneration of adult cells. We concluded that aspernolide A induced the apoptosis of laryngeal cancer Hep-2 and TU212 cells by activating the apoptotic signaling pathway in mitochondria and inhibiting the phosphorylation of STAT3, and cyclin was also involved (Figure 8). Our results are helpful to the current study of aspernolide A and provide evidence for its potential use as an antineoplastic agent.

## Supplementary Materials

The following are available online, Figure S1: ^1^H-NMR spectrum (600 MHz, d6-acetone) of aspernolide A, Figure S2: ^13^C-NMR spectrum (150 MHz, d6-acetone) of aspernolide A.
