# Supplementary material for: Aspernolide A Inhibits the Proliferation of Human Laryngeal Carcinoma Cells through the Mitochondrial Apoptotic and STAT3 Signaling Pathways
Source: Molecules. 2019 Mar 19;24(6):1074. doi: 10.3390/molecules24061074 (PMC6471715; doi:10.3390/molecules24061074)

Supporting Information

Figure S1. ^{1}H NMR spectrum (600 MHz, *d_6-acetone*) of aspernolide A.

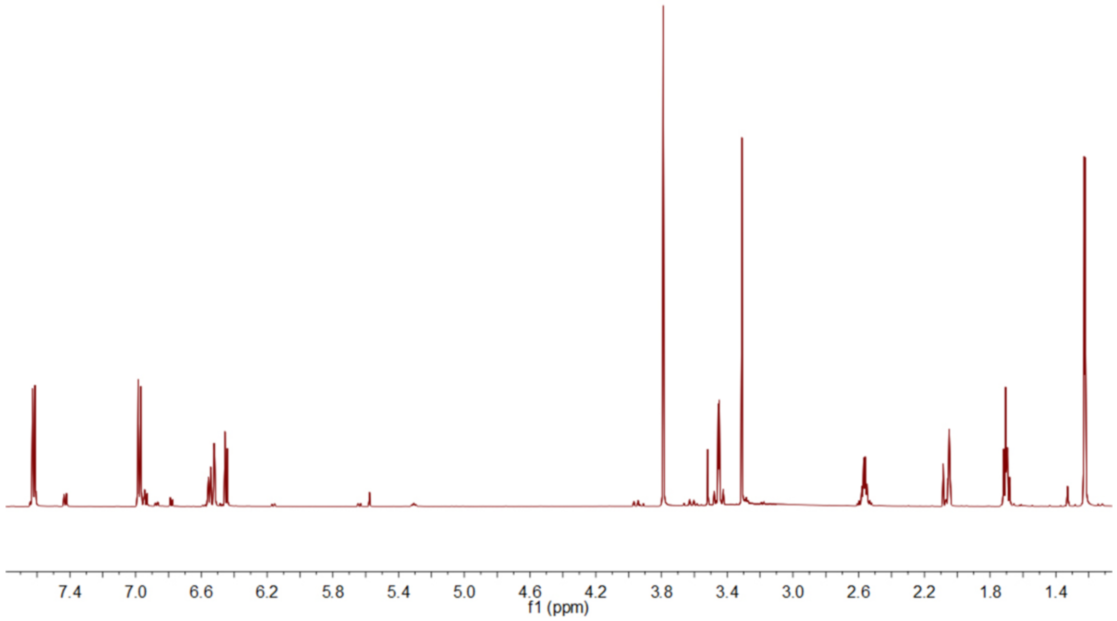

Figure S2. ^{13}C NMR spectrum (150 MHz, *d6-acetone*) of aspernolide A.

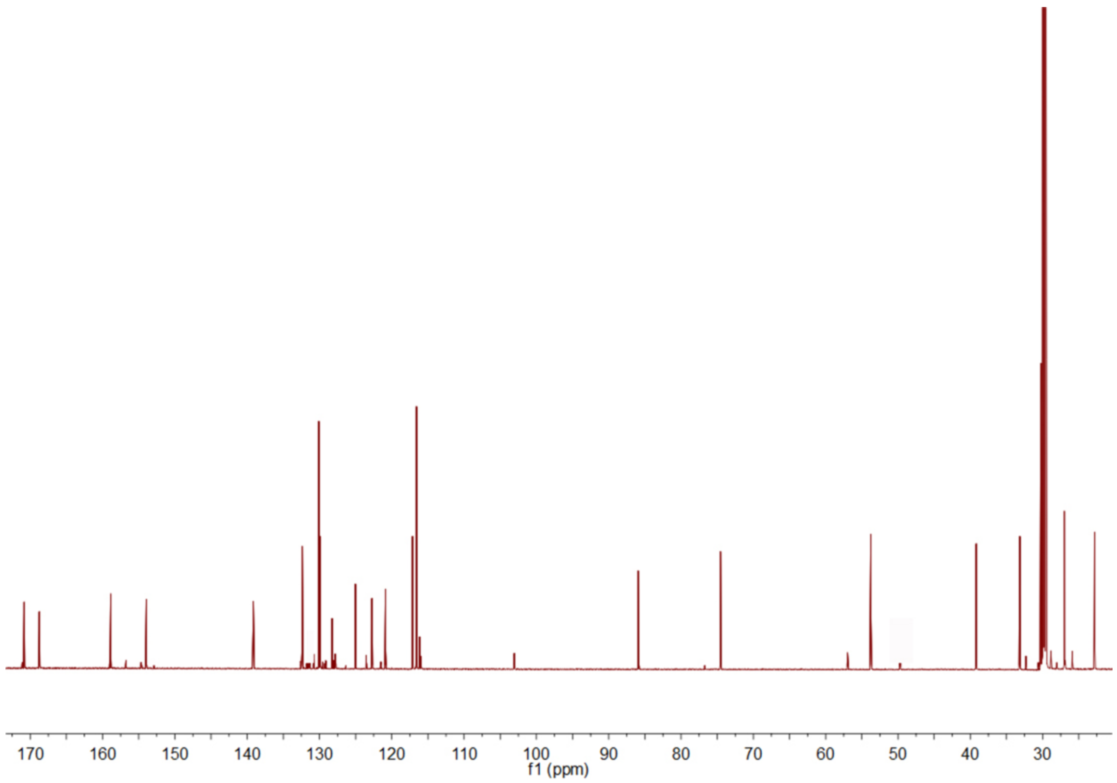

Supplement: Supplementary file 1 [file molecules-24-01074-s001.pdf]
